# Comorbidity between Type 2 Diabetes and Depression in the Adult Population: Directions of the Association and Its Possible Pathophysiological Mechanisms

**DOI:** 10.1155/2015/164760

**Published:** 2015-09-17

**Authors:** Line Iden Berge, Trond Riise

**Affiliations:** ^1^Department of Global Public Health and Primary Care, University of Bergen, 5018 Bergen, Norway; ^2^Psychiatric Division, Bergen University Hospital, 5021 Bergen, Norway

## Abstract

Type 2 diabetes and depression are regarded as comorbid conditions, and three possible directions of the association between the diseases can underlie this observation of comorbidity. First, common etiology can increase a person's risk of both diseases; second, persons with type 2 diabetes have increased prevalence or risk of future development of depression; or third, persons with depression have increased prevalence or risk of development of type 2 diabetes. This review gives an overview over possible pathophysiological mechanisms for each of the directions of the association between type 2 diabetes and depression and further discusses epigenetics as an additional, direction independent approach. We argue that unspecific pathophysiological mechanisms involved in the stress response might, at least to some extent, explain each of the directions of the association between type 2 diabetes and depression, while changes in brain structure and function among persons with diabetes and possible increased risk of development of type 2 diabetes after use of antidepressant agents could represent more disease specific mechanisms underlying the comorbidity.

## 1. Background

The term comorbidity was introduced in 1970 by Feinstein, a doctor of internal medicine and epidemiologist who studied cooccurrence of diseases among persons with diabetes [1]. He suggested comorbidity to be defined as “any distinct clinical entity that has existed or that may occur during the course of a patient who has the index disease under study,” in which “index disease” is understood as the main condition under study [1]. Later, it has been recommended to reserve the term comorbidity to conditions that are not known as a direct consequence of the index disease, while use of the term complication should imply a strong evidence of causality [2]. Today, comorbidity is often used to describe the cooccurrence of separate distinct diseases at one specific point of time, regardless of the direction between them, and when the association observed is not expected to be explained by causality [3].

**Figure 1:**
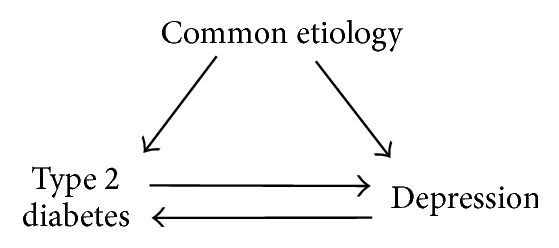
Possible directions of the association between type 2 diabetes and depression.

The International Diabetes Federation estimates the global prevalence of diabetes in 2013 to approximate 8% [4], while The World Mental Health Survey undertaken from 2001 to 2007 found lifetime and 12-month prevalence of major depressive episode to reach 15% and 6%, respectively [5]. Major depression and diabetes are ranked the 4th and 9th most important causes of disability adjusted life years (DALYS) in the developed world [6], reflecting the high prevalence and, for particularly depression, the early onset of disease. Further, the effect of comorbid diabetes and depression on decrements in health has been shown to be interactive, suggesting a negative effect on health beyond that expected by adding the effect of the two disorders [7]. Perhaps due to this large impact on public health, numerous studies have investigated various aspects of the comorbidity between depression and diabetes, in particular type 2 diabetes. For instance, previous work has found evidence of a bidirectional association between type 2 diabetes and depression [8–11]. As outlined in Figure 1, three possible directions of the association between type 2 diabetes and depression can underlie the observation of comorbidity between the disorders. First, common etiology can increase a person's risk of both diseases; second, persons with type 2 diabetes have increased prevalence or risk of future development of depression; or third, persons with depression have increased prevalence or risk of development of type 2 diabetes.

Pathophysiology is defined as the study of mechanisms of disease, that is, how changes in normal physiology can cause diseases [12]. Some literatures have in general terms sought to describe the pathophysiology underlying the comorbidity between type 2 diabetes and depression [13–16]; however, to our best knowledge, no previous review has addressed possible direction specific pathophysiological mechanisms for why we observe comorbidity between type 2 diabetes and depression. This review therefore aims to give an overview over possible pathophysiological mechanisms for each of the directions of the association between type 2 diabetes and depression, with special emphasis on stress-theory. In this review, the term “depression” not necessarily equals the diagnostic criteria for various affective disorders according to DSM-V or ICD-10 but should rather be understood broadly as a heterogeneous group of disorders with core symptoms of impaired mood and lack of interest and energy.

## 2. Common Etiology: Possible Pathophysiological Mechanisms

Cooccurrence of type 2 diabetes and depression more often than expected by chance can be a result of common etiology, overall considered to be either shared genetic or environmental factors.

### 2.1. Genetic Factors

Theoretically, comorbidity between type 2 diabetes and depression could be explained by common genetic factors; that is, genes associated with one of the disorders could be associated with or inherited together with genes associated with the other disorder. This is however not supported by the literature. Most likely due to large heterogeneity of the disorders, no previous genome wide association studies (GWAS) have explored the comorbidity between type 2 diabetes and depression, while only one twin study has investigated the comorbidity between depression and diabetes, types 1 and 2 combined [17]. In this latter study including about 1200 middle-aged male twins, about 50 percent homo- and heterozygote, respectively, the estimated genetic correlation between depression and diabetes was *r* = 0.19 (95% CI: 0.00–0.46). The authors concluded that they found overall little evidence of common genetic factors accounting for the comorbidity between diabetes and depression among middle-aged men.

### 2.2. Environmental Factors

A range of environmental factors is associated with both type 2 diabetes and depression, and it is likely that these factors can explain at least part of the association between the diseases. Examples of such factors include, but are not limited to, unfavorable lifestyle such as inactivity, poor sleep, and diet [18–20], psychological and social factors including early life stress [21, 22] and demanding socioeconomic position [23, 24], and more strict “medical” factors such as other conditions (e.g., obesity) [25, 26] and medical treatment (e.g., corticosteroids) [27]. As an example, early life stress in terms of evacuation abroad to temporary foster care during World War 2 has later in life been associated with increased risk of type 2 diabetes (odds ratio 1.4 (95% CI: 1.1, 1.9)) [21], as well as increased risk of depressive symptoms (Beck Depression Inventory score ≥ 10) (odds ratio 1.7 (95% CI: 1.1, 2.6)) [22]. Further, a meta-analysis from 2011 showed that low socioeconomic position measured in terms of education, income, and occupational status was associated with increased risk of type 2 diabetes relative to high status [23] (RR education 1.41 (95% CI: 1.28, 1.51), RR occupation 1.31 (95% CI: 1.09, 1.57), and RR income 1.40 (95% CI: 1.05, 1.88)), while cohort studies have found up to a doubled risk of depression among children of parents with low socioeconomic status compared with children of parents with high status [24]. As both type 2 diabetes and depression are highly prevalent diseases in the developed world, the absolute effect of this relative increased risk at population level is particularly high.

**Figure 2:**
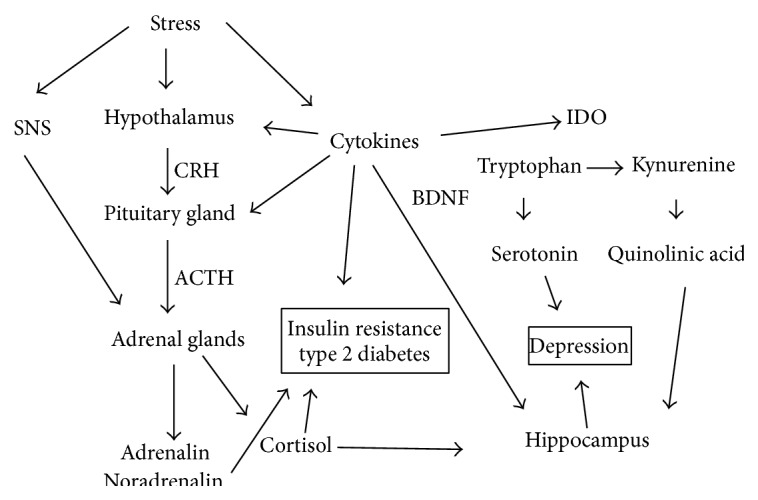
The stress response and possible pathophysiological mechanisms contributing to type 2 diabetes and depression.

The common denominator for these environmental factors is that they represent a threat to the homeostasis of the stress systems of the body. To a large extent, the body is able to regulate the effect of acute stress, mainly via the hormone system, the autonomous system, and the inflammatory system [28, 29]. However, if these systems are highly activated over time, the stress response may become unintentional and eventually harmful [28]. In the following section and further illustrated in Figure 2, we outline possible pathophysiological mechanisms illustrating how chronic stress responses can increase the risk of development of both type 2 diabetes and depression.

Stress activates the hypothalamus pituitary adrenal axis, often abbreviated HPA-axis, increasing the production of cortisol from the adrenal cortex [28, 30]. Additionally, stress increases stimulation of the sympathetic part of the autonomous nervous system (SNS), further increasing the production of adrenalin and noradrenalin from the adrenal medulla [28, 30]. It is well established that these stress hormones exert an overall catabolic effect on the body by mobilizing glucose as well as fat and proteins to the blood [28], counteracting the effect of insulin which mainly acts as an anabolic hormone facilitating uptake and storage of glucose. This represents a mechanism by which chronic stress activation can lead to insulin resistance and eventually type 2 diabetes.

Either directly, or via the HPA-axis and the autonomous nervous system [31], the inflammation system is additionally activated by stress, evident by increased production of proinflammatory cytokines from immune cells and tissues, such as Il-6, TNF-*α*, and interferon-*γ* [32, 33]. The cytokines can directly stimulate receptors in the hypothalamus and the pituitary, thereby facilitating more production of cortisol [34]. It has further been suggested that proinflammatory cytokines can contribute to development of insulin resistance directly by modulation of the insulin receptor in periphery tissues. For example, Grimble proposes in a review in 2002 over inflammatory status and insulin resistance that TNF-*α*, via reduced phosphorylation of a subunit of the insulin receptor, contribute to development of insulin resistance [35]. Population based studies have further underlined the importance of inflammatory factors in the development of type 2 diabetes. In the British Whitehall II Study, the association between low socioeconomic status and risk of incident type 2 diabetes was attenuated by up to one-third after adjustment for inflammatory factors measured during follow-up [36].

In addition to the contribution of cortisol and cytokines on development of insulin resistance, they are proposed to also have a negative impact on areas or systems in the brain associated with alterations in depression, such as the monoamine system and hippocampus [37, 38]. First, hippocampus exhibits glucocorticoid receptors, and it has been demonstrated that stimulation over time with high levels of cortisol can result in reduced neurogenesis and synaptic plasticity as well as apoptosis in distinct areas of hippocampus [39]. Second, an effect of cytokines on hippocampus mediated by increased levels of oxidative stress resulting in lower levels of BDNF (brain derived neurotropic factor) has been proposed [40]. BDNF is a neurotropic peptide essential for survival and proliferation of neurons in the brain, and low levels have been found among persons with depression, while use of antidepressant agents has been associated with upregulation of BDNF production [37, 38]. Finally, it has been proposed that certain proinflammatory cytokines can increase degradation of tryptophan, an essential amino acid [41]. This degradation takes place in the kynurenine pathway mainly in liver, kidneys, and immune cells. It has been shown that cytokines induce one rate limiting enzyme in this pathway, the indoleamine dioxygenase (IDO), while cortisol can upregulate another rate limiting enzyme, tryptophan 2,3-dioxygenase (TDO), resulting in lower concentrations of available tryptophan and higher concentrations of other metabolites in the kynurenine pathway [41]. Serotonin, maybe the most important monoamine implicated in the pathophysiology of depression [37, 38], can solely be produced by tryptophan, and theories exist that low concentration of tryptophan consequently will result in less available serotonin in the brain [42]. Moreover, some of the additional metabolites further down the kynurenine pathway, such as quinolinic acid, are suggested to have an additional negative impact on hippocampus in terms of reduced neurogenesis and induction of apoptosis [41]. An overview over theories on possible pathophysiological mechanisms linking cytokines with depression can be found in a review by Myint and Kim from 2014 [41].

In summary, this hypothesis on chronic and unintentional stress response is a suggestion on how common etiology in terms of environmental factors via distinct pathophysiological mechanisms possibly can contribute to the development of both type 2 diabetes and depression.

## 3. Persons with Type 2 Diabetes Have Increased Prevalence and Risk of Depression: Possible Pathophysiological Mechanisms

Several population based studies have established that persons with type 2 diabetes have both increased prevalence and incidence of depression relative to persons without diabetes [43, 44]. While Ali et al. found an odds ratio of 1.6 (95% CI: 1.2, 2.0) for comorbid depression among persons with type 2 diabetes in a meta-analysis of cross-sectional studies [43], Nouwen et al. showed a relative risk of 1.24 (95% CI: 1.09, 1.40) for incident depression among persons with type 2 diabetes in a meta-analysis of prospective studies [44]. How can these findings be explained in terms of pathophysiological mechanisms?

First, it is likely that several unspecific factors associated with chronic diseases can increase the risk of depression. One example of such factor can be the psychological burden of being ill. It is easy to imagine it being demanding to maintain stable blood glucose over time, to monitor glucose levels and calculate dosage of medication. Further, type 2 diabetes is often associated with an unfavorable lifestyle in terms of inactivity, overweight, and obesity [19]. In a large population based, cross-sectional study from Norway, persons with both types 1 and 2 diabetes reported lower levels of activity and had higher BMI than persons without diabetes [45]. These psychological and lifestyle factors can again be viewed as a type of unspecific stress threatening the homeostasis of the body and therefore possibly increase the risk of depression via the pathophysiological mechanisms outlined earlier.

Second, the majority of persons with type 2 diabetes need oral antidiabetic medication to maintain stable blood sugar within reference range [46]. Hypothetically, increased prevalence and risk of depression could be caused by antidiabetic treatment. According to the literature, there is little evidence that depression is a plausible side effect of insulin, biguanides, and/or sulfonylurea. However, use of insulin and sulfonylureas is associated with weight gain [47], and if one views weight gain and possibly overweight and obesity as a type of unspecific stress, the pathophysiological mechanisms addressing how stress can cause depression might also be relevant in this context.

Third, diabetes is associated with structural changes in certain areas of the brain. A systematic review over brain imaging studies on persons with diabetes from 2006 concluded that diabetes was associated with cerebral atrophy and lacunar infarcts, as well as regional alterations in cerebral blood flow [48]. A more recent study on middle-aged persons with type 2 diabetes of less than 10-year duration showed reductions in brain volume restricted to the hippocampus relative to controls with similar age, sex, and level of education [49]. In multivariate regression analysis, an inverse association between HbA1c and hippocampal volume was found, particularly interesting since the patients had fairly well controlled diabetes with a mean HbA1c of 6.9% [49]. The finding of an inverse association between HbA1c and structural changes in hippocampus has also been confirmed in studies of patients with type 1 diabetes [50], supporting a theory of a possible effect of glucose levels on the brain. When compared to a nondiabetic control group, persons with well controlled type 1 diabetes aged 25–40 years without complications showed lower density of gray matter in several brain regions, including hippocampus [50]. Further, an inverse association with lower density and higher HbA1c and number of severe hypoglycemic events were found, leading the authors to conclude that persistent hyperglycemia and severe hypoglycemia likely have impact on brain structure [50].

Further, a research group from US has conducted several studies comparing changes in brain structure, function, and biochemistry among persons with type 2 diabetes and the combination of type 2 diabetes and depression with healthy controls [51–53]. For several outcomes, such as cortical gray matter thickness and level of the excitatory neurotransmitter glutamate, the most pronounced effect was found among persons with both type 2 diabetes and depression, and the authors consequently suggest that “there is a strong subcortical neurobiological component to depression in patients with type 2 diabetes” [53].

## 4. Persons with Depression Have Increased Prevalence and Risk of Type 2 Diabetes: Possible Pathophysiological Mechanisms

Finally, we might observe comorbidity between type 2 diabetes and depression because persons with depression have increased prevalence [54] and incidence of type 2 diabetes [55]. Interestingly, the association has in several studies tended to be stronger when considering depression as the exposure and diabetes as the outcome than the other way around [56, 57]. For example, a meta-analysis exploring this bidirectional association found a relative risk of 1.60 (95% CI: 1.37, 1.88) for type 2 diabetes among persons with depression, while persons with type 2 diabetes had a modest increased risk of depression with an relative risk of 1.15 (95% CI: 1.02, 1.30) [9].

Obviously, psychological and lifestyle factors associated with depression can again be viewed as a type of stress and increase the risk of type 2 diabetes via the pathophysiological mechanisms outlined earlier. Illustrating the negative impact of depression on insulin resistance even in persons free of diabetes, Asghar et al. have shown that the impaired insulin sensitivity among women with major depression was improved after successful treatment with antidepressant agents [58].

Potential risk of diabetes after use of antidepressant agents is a hot topic in the literature on comorbidity between diabetes and depression. A systematic review on antidepressant medication as a risk factor for type 2 diabetes and impaired glucose regulation was published in 2013 in Diabetes Care [59]. The authors sought to conduct a meta-analysis, but this was not feasible due to large heterogeneity and discrepancies in quality between the single studies. The findings were mixed; use of some antidepressants, particularly those with strong noradrenergic properties, was associated with worsening of glucose control, while use of other agents in fact was associated with improved glycemic control, such as selective serotonin reuptake inhibitors. Additionally, a meta-analysis on antidepressant use and risk of diabetes was published in 2013 in Korean Journal of Family Medicine [60], including a majority of the studies included in the review in Diabetes Care. Stratified analyses on type of antidepressant agents showed increased risk of type 2 diabetes after use of both selective serotonin reuptake inhibitors (SSRI) and tricyclic antidepressant agents (TCAs) (relative risk SSRI: 1.35 (95% CI: 1.15, 1.58), relative risk TCA 1.57 (95% CI: 1.26, 1.96)). Further analyses adjusted for BMI, severity of depression, and physical activity showed a significantly increased risk of type 2 diabetes of 14% (RR: 1.14 (95% CI: 1.01, 1.28)).

Animal studies have shown that, for example, selective serotonin reuptake inhibitors directly can inhibit both the production and the effect of insulin [61, 62]. Cultivating hepatic rat cells incubated with paroxetine and sertraline, Israeli researchers found weaker response of insulin on the insulin receptor, an effect mediated via reduced phosphorylation of some of the subunits within the receptor complex [62]. A few years later, the same group demonstrated that incubating beta-cells from rat pancreas with paroxetine, sertraline, and fluoxetine attenuated the production of insulin and in some of the samples induced apoptosis of the beta-cells [61]. If use of antidepressant agents independently of weight gain and severity of depression increases the risk of type 2 diabetes, this direct effect on the insulin receptor could be one of the mechanisms possibly explaining it.

## 5. Direction Independent Approach: Epigenetics

Epigenetics is the study of factors influencing the long term expression of the genes, that is, how one set of genes can be transcribed differently depending on environmental factors [63]. These changes in transcription are stable over time and can be inherited [63]. In brief, the change is facilitated via methylation of the DNA or via modification of the histones, around which the DNA is packed [63]. It has been suggested that epigenetic changes, at least in theory, can contribute to the occurrence of comorbidity between diseases [64]. Epigenetics could therefore represent a new element added to each of the three presented theories for why we observe comorbidity between type 2 diabetes and depression and should consequently be viewed as a direction independent approach.

To our best knowledge, no study has addressed if or how epigenetic changes might explain the established comorbidity between type 2 diabetes and depression; however, studies have documented epigenetic changes after stress [65] and the changes in epigenetics can be of importance in the pathogenesis of both type 2 diabetes [66] and depression [65]. Alterations in gene expression were found in postmortem specimens from persons with type 2 diabetes compared to controls without diabetes matched on age, in addition to corresponding changes in expression of synaptic proteins [67]. The authors suggested that such studies might elucidate possible epigenetic mechanisms explaining the increased risk of dementia among persons with diabetes; however, one might argue that it could shed light on possible epigenetic mechanisms explaining the increased prevalence and incidence of depression among persons with diabetes as well.

## 6. Limitations

Due to the diversity of the reviewed studies, the term “depression” not necessarily equals the diagnostic criteria for various affective disorders according to DSM-V or ICD-10 in this review but should rather be understood broadly as a heterogeneous group of disorders with core symptoms of impaired mood and lack of interest and energy. This understanding of depression represents a limitation, mainly because we cannot exclude that symptoms interpreted as depression in this review rather are symptoms of another psychiatric disorder, for example, anxiety disorders. Additionally, it limits us from exploring the association between type 2 diabetes and the severity of depression and consequently further limits a discussion on how this could be related to the various pathophysiological mechanisms.

## 7. Conclusion

Several different pathophysiological mechanisms can underlie the comorbidity between type 2 diabetes and depression, most likely interacting with each other, thereby possibly reinforcing a negative effect on the development of the diseases. However, the unspecific mechanisms involved in the stress response might, at least to some extent, explain each of the directions of the association between type 2 diabetes and depression. In order to determine the impact of the stress response due to common etiology at population level, a prospective study on the association between type 2 diabetes and depression adjusting for information on various forms of stressors such as lifestyle, psychological, and social factors early in life could be conducted. To determine the contribution of the different pathophysiological mechanisms at person level, the best approach is probably to first determine the temporal sequence between the disorders.

## References

[1] Feinstein A. R. (1970). The pre-therapeutic classification of co-morbidity in chronic disease. *Journal of Chronic Diseases*.

[2] Ording A. G., Sørensen H. T. (2013). Concepts of comorbidities, multiple morbidities, complications, and their clinical epidemiologic analogs. *Clinical Epidemiology*.

[3] Berge L. I. (2014). *Depression and Migraine Comorbid to Diabetes. Epidemiological Studies Utilizing Data from the Norwegian Prescription Database and the Hordaland Health Stusy*.

[4] International Diabetes Federation (2013). *Atlas*.

[5] Bromet E., Andrade L. H., Hwang I. (2011). Cross-national epidemiology of DSM-IV major depressive episode. *BMC Medicine*.

[6] National Institute of Health Meterics Evaluation Global Burden of Disease.

[7] Moussavi S., Chatterji S., Verdes E., Tandon A., Patel V., Ustun B. (2007). Depression, chronic diseases, and decrements in health: results from the World Health Surveys. *The Lancet*.

[8] Golden S. H., Lazo M., Carnethon M. (2008). Examining a bidirectional association between depressive symptoms and diabetes. *Journal of the American Medical Association*.

[9] Mezuk B., Eaton W. W., Albrecht S., Golden S. H. (2008). Depression and type 2 diabetes over the lifespan: a meta-analysis. *Diabetes Care*.

[10] Pan A., Lucas M., Sun Q. (2010). Bidirectional association between depression and type 2 diabetes mellitus in women. *Archives of Internal Medicine*.

[11] Renn B. N., Feliciano L., Segal D. L. (2011). The bidirectional relationship of depression and diabetes: a systematic review. *Clinical Psychology Review*.

[12] Huether S. E., McCance K. L. (2013). *Understanding Pathophysiology*.

[13] Champaneri S., Wand G. S., Malhotra S. S., Casagrande S. S., Golden S. H. (2010). Biological basis of depression in adults with diabetes. *Current Diabetes Reports*.

[14] Golden S. H. (2007). A review of the evidence for a neuroendocrine link between stress, depression and diabetes mellitus. *Current Diabetes Reviews*.

[15] Katon M. S. (2010). *Depression and Diabetes*.

[16] Rustad J. K., Musselman D. L., Nemeroff C. B. (2011). The relationship of depression and diabetes: pathophysiological and treatment implications. *Psychoneuroendocrinology*.

[17] Scherrer J. F., Xian H., Lustman P. J. (2011). A test for common genetic and environmental vulnerability to depression and diabetes. *Twin Research and Human Genetics*.

[18] Nanri A. (2013). Nutritional epidemiology of type 2 diabetes and depressive symptoms. *Journal of Epidemiology*.

[19] Reis J. P., Loria C. M., Sorlie P. D., Park Y., Hollenbeck A., Schatzkin A. (2011). Lifestyle factors and risk for new-onset diabetes: a population-based cohort study. *Annals of Internal Medicine*.

[20] Berk M., Sarris J., Coulson C. E., Jacka F. N. (2013). Lifestyle management of unipolar depression. *Acta Psychiatrica Scandinavica. Supplementum*.

[21] Alastalo H., Räikkönen K., Pesonen A.-K. (2009). Cardiovascular health of Finnish war evacuees 60 years later. *Annals of Medicine*.

[22] Pesonen A.-K., Räikkönen K., Heinonen K., Kajantie E., Forsén T., Eriksson J. G. (2007). Depressive symptoms in adults separated from their parents as children: a natural experiment during World War II. *American Journal of Epidemiology*.

[23] Agardh E., Allebeck P., Hallqvist J., Moradi T., Sidorchuk A. (2011). Type 2 diabetes incidence and socio-economic position: a systematic review and meta-analysis. *International Journal of Epidemiology*.

[24] Gilman S. E., Kawachi I., Fitzmaurice G. M., Buka S. L. (2002). Socioeconomic status in childhood and the lifetime risk of major depression. *International Journal of Epidemiology*.

[25] Menke A., Rust K. F., Fradkin J., Cheng Y. J., Cowie C. C. (2014). Associations between trends in race/ethnicity, aging, and body mass index with diabetes prevalence in the United States: a series of cross-sectional studies. *Annals of Internal Medicine*.

[26] Luppino F. S., de Wit L. M., Bouvy P. F. (2010). Overweight, obesity, and depression: a systematic review and meta-analysis of longitudinal studies. *Archives of General Psychiatry*.

[27] National Health Services England http://www.nhs.uk/Conditions/Corticosteroid-(drugs)/Pages/Sideeffects.aspx.

[28] Charmandari E., Tsigos C., Chrousos G. (2005). Endocrinology of the stress response. *Annual Review of Physiology*.

[29] Black P. H. (2002). Stress and the inflammatory response: a review of neurogenic inflammation. *Brain, Behavior, and Immunity*.

[30] Chrousos G. P., Gold P. W. (1992). The concepts of stress and stress system disorders: overview of physical and behavioral homeostasis. *The Journal of the American Medical Association*.

[31] Elenkov I. J., Wilder R. L., Chrousos G. P., Vizi E. S. (2000). The sympathetic nerve—an integrative interface between two supersystems: the brain and the immune system. *Pharmacological Reviews*.

[32] Maes M., Song C., Lin A. (1998). The effects of psychological stress on humans: increased production of pro-inflammatory cytokines and a Th1-like response in stress-induced anxiety. *Cytokine*.

[33] Goebel M. U., Mills P. J., Irwin M. R., Ziegler M. G. (2000). Interleukin-6 and tumor necrosis factor-alpha production after acute psychological stress, exercise, and infused isoproterenol: differential effects and pathways. *Psychosomatic Medicine*.

[34] Raison C. L., Capuron L., Miller A. H. (2006). Cytokines sing the blues: inflammation and the pathogenesis of depression. *Trends in Immunology*.

[35] Grimble R. F. (2002). Inflammatory status and insulin resistance. *Current Opinion in Clinical Nutrition and Metabolic Care*.

[36] Stringhini S., da Batty G. D., Bovet P. (2013). Association of lifecourse socioeconomic status with chronic inflammation and type 2 diabetes risk: the Whitehall II prospective cohort study. *PLoS Medicine*.

[37] Belmaker R. H., Agam G. (2008). Major depressive disorder. *The New England Journal of Medicine*.

[38] Rot M. A. H., Mathew S. J., Charney D. S. (2009). Neurobiological mechanisms in major depressive disorder. *Canadian Medical Association Journal*.

[39] de Kloet E. R., Joëls M., Holsboer F. (2005). Stress and the brain: from adaptation to disease. *Nature Reviews Neuroscience*.

[40] Anisman H. (2009). Cascading effects of stressors and inflammatory immune system activation: implications for major depressive disorder. *Journal of Psychiatry & Neuroscience*.

[41] Myint A.-M., Kim Y.-K. (2014). Network beyond IDO in psychiatric disorders: revisiting neurodegeneration hypothesis. *Progress in Neuro-Psychopharmacology and Biological Psychiatry*.

[42] Neumeister A. (2003). Tryptophan depletion, serotonin, and depression: where do we stand?. *Psychopharmacology Bulletin*.

[43] Ali S., Stone M. A., Peters J. L., Davies M. J., Khunti K. (2006). The prevalence of co-morbid depression in adults with Type 2 diabetes: a systematic review and meta-analysis. *Diabetic Medicine*.

[44] Nouwen A., Winkley K., Twisk J. (2010). Type 2 diabetes mellitus as a risk factor for the onset of depression: a systematic review and meta-analysis. *Diabetologia*.

[45] Engum A., Mykletun A., Midthjell K., Holen A., Dahl A. A. (2005). Depression and diabetes: a large population-based study of sociodemographic, lifestyle, and clinical factors associated with depression in type 1 and type 2 diabetes. *Diabetes Care*.

[46] Claudi T., Ingskog W., Cooper J. G., Jenum A. K., Hausken M. F. (2008). Quality of diabetes care in Norwegian general practice. *Tidsskrift for den Norske Laegeforening*.

[47] Turner R., UK Prospective Diabetes Study (UKPDS) Group (1998). Intensive blood-glucose control with sulphonylureas or insulin compared with conventional treatment and risk of complications in patients with type 2 diabetes (UKPDS 33). *The Lancet*.

[48] van Harten B., de Leeuw F.-E., Weinstein H. C., Scheltens P., Biessels G. J. (2006). Brain imaging in patients with diabetes: a systematic review. *Diabetes Care*.

[49] Gold S. M., Dziobek I., Sweat V. (2007). Hippocampal damage and memory impairments as possible early brain complications of type 2 diabetes. *Diabetologia*.

[50] Musen G., In K. L., Sparks C. R. (2006). Effects of type 1 diabetes on gray matter density as measured by voxel-based morphometry. *Diabetes*.

[51] Ajilore O., Haroon E., Kumaran S. (2007). Measurement of brain metabolites in patients with type 2 diabetes and major depression using proton magnetic resonance spectroscopy. *Neuropsychopharmacology*.

[52] Ajilore O., Narr K., Rosenthal J. (2010). Regional cortical gray matter thickness differences associated with type 2 diabetes and major depression. *Psychiatry Research—Neuroimaging*.

[53] Kumar A., Gupta R., Thomas A., Ajilore O., Hellemann G. (2009). Focal subcortical biophysical abnormalities in patients diagnosed with type 2 diabetes and depression. *Archives of General Psychiatry*.

[54] Holt R. I. G., Phillips D. I. W., Jameson K. A., Cooper C., Dennison E. M., Peveler R. C. (2009). The relationship between depression and diabetes mellitus: findings from the Hertfordshire Cohort Study. *Diabetic Medicine*.

[55] Knol M. J., Twisk J. W. R., Beekman A. T. F., Heine R. J., Snoek F. J., Pouwer F. (2006). Depression as a risk factor for the onset of type 2 diabetes mellitus. A meta-analysis. *Diabetologia*.

[56] Rotella F., Mannucci E. (2013). Diabetes mellitus as a risk factor for depression. A meta-analysis of longitudinal studies. *Diabetes Research and Clinical Practice*.

[57] Rotella F., Mannucci E. (2013). Depression as a risk factor for diabetes: a meta-analysis of longitudinal studies. *The Journal of Clinical Psychiatry*.

[58] Asghar S. M. A., Hussain A., Diep L., Bhowmik B., Thorsby P. (2012). Depression and insulin resistance in non-diabetic subjects: an intervention study with insulin clamp techniqe. *International Journal of Clinical Medicine*.

[59] Barnard K., Peveler R. C., Holt R. I. G. (2013). Antidepressant medication as a risk factor for type 2 diabetes and impaired glucose regulation. *Diabetes Care*.

[60] Yoon J. M., Cho E.-G., Lee H.-K., Park S. M. (2013). Antidepressant use and diabetes mellitus risk: a meta-analysis. *Korean Journal of Family Medicine*.

[61] Isaac R., Boura-Halfon S., Gurevitch D., Shainskaya A., Levkovitz Y., Zick Y. (2013). Selective serotonin reuptake inhibitors (SSRIs) inhibit insulin secretion and action in pancreatic beta cells. *The Journal of Biological Chemistry*.

[62] Levkovitz Y., Ben-shushan G., Hershkovitz A. (2007). Antidepressants induce cellular insulin resistance by activation of IRS-1 kinases. *Molecular and Cellular Neuroscience*.

[63] Holliday R. (2006). Epigenetics: a historical overview. *Epigenetics*.

[64] Jakovljević M., Reiner Ž., Miličić D., Crnčević Ž. (2010). Comorbidity, multimorbidity and personalized psychosomatic medicine: epigenetics rolling on the horizon. *Psychiatria Danubina*.

[65] Nestler E. J. (2014). Epigenetic mechanisms of depression. *JAMA Psychiatry*.

[66] Dayeh T., Volkov P., Salö S. (2014). Genome-wide DNA methylation analysis of human pancreatic islets from type 2 diabetic and non-diabetic donors identifies candidate genes that influence insulin secretion. *PLoS Genetics*.

[67] Wang J., Gong B., Zhao W. (2014). Epigenetic mechanisms linking diabetes and synaptic impairments. *Diabetes*.

